# Pyromellitic acid–sarcosine (1/2)

**DOI:** 10.1107/S1600536808009045

**Published:** 2008-04-10

**Authors:** Sérgio R. Domingos, Manuela Ramos Silva, Nuno D. Martins, Ana Matos Beja, J. A. Paixão

**Affiliations:** a CEMDRX, Physics Department, University of Coimbra, P-3004-516 Coimbra, Portugal

## Abstract

The title compound, C_10_H_6_O_8_·2C_3_H_7_NO_2_, crystallizes as an adduct with the acid and amino acid mol­ecules in their neutral forms. The asymmetric unit contains one half of a centrosymmetric pyromellitic acid mol­ecule and one sarcosine mol­ecule. The sarcosine has the amine group protonated and the carboxyl group deprotonated, as is usual for amino acids (zwitterionic form). The pyromellitic acid mol­ecules retain the four carboxyl H atoms with the carboxyl groups rotated out of the ring plane [O—C—C—C torsion angles = 24.1 (3) and 61.6 (2)°]. There is a three-dimensional hydrogen-bond network linking the mol­ecules.

## Related literature

For related compounds, see: Yaghi *et al.* (1997 ▶); Arora & Pedireddi (2003 ▶); Rochon & Massarweh (2001 ▶); Kumagai *et al.* (2003 ▶).
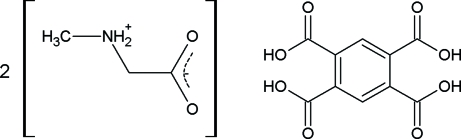


## Experimental

### 

#### Crystal data

C_10_H_6_O_8_·2C_3_H_7_NO_2_
*M* *_r_* = 432.34
Monoclinic, 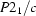

*a* = 8.8894 (3) Å
*b* = 5.4118 (2) Å
*c* = 20.2205 (7) Å
β = 104.388 (2)°
*V* = 942.25 (6) Å^3^
*Z* = 2
Mo *K*α radiation
μ = 0.13 mm^−1^
*T* = 293 (2) K
0.47 × 0.10 × 0.07 mm

#### Data collection

Bruker APEX CCD area-detector diffractometer
Absorption correction: multi-scan (*SADABS*; Sheldrick, 2000 ▶) *T*_min_ = 0.915, *T*_max_ = 0.998
16656 measured reflections
2351 independent reflections
1643 reflections with *I* > 2σ(*I*)
*R*_int_ = 0.047

#### Refinement

*R*[*F* ^2^ > 2σ(*F* ^2^)] = 0.041
*wR*(*F* ^2^) = 0.115
*S* = 1.01
2351 reflections
166 parameters
Only H-atom coordinates refined
Δρ_max_ = 0.24 e Å^−3^
Δρ_min_ = −0.21 e Å^−3^

### 

Data collection: *SMART* (Bruker, 2003 ▶); cell refinement: *SAINT-Plus* (Bruker, 2003 ▶); data reduction: *SAINT-Plus* (Bruker, 2003 ▶); program(s) used to solve structure: *SHELXS97* (Sheldrick, 2008 ▶); program(s) used to refine structure: *SHELXL97* (Sheldrick, 2008 ▶); molecular graphics: *ORTEPIII* (Burnett & Johnson, 1996 ▶), *ORTEP-3 for Windows* (Farrugia, 1997 ▶) and *PLATON* (Spek, 2003 ▶); software used to prepare material for publication: *SHELXL97*.

## Supplementary Material

Crystal structure: contains datablocks global, I. DOI: 10.1107/S1600536808009045/dn2326sup1.cif

Structure factors: contains datablocks I. DOI: 10.1107/S1600536808009045/dn2326Isup2.hkl

Additional supplementary materials:  crystallographic information; 3D view; checkCIF report

## Figures and Tables

**Table 1:** Hydrogen-bond geometry (Å, °)

*D*—H⋯*A*	*D*—H	H⋯*A*	*D*⋯*A*	*D*—H⋯*A*
O2—H2⋯O5^i^	0.93 (2)	1.61 (2)	2.5216 (18)	166 (2)
O3—H3⋯O5	0.98 (2)	1.62 (2)	2.6026 (18)	179 (2)
N1—H1*A*⋯O6^ii^	0.87 (2)	2.11 (2)	2.854 (2)	143.4 (17)
N1—H1*A*⋯O1^iii^	0.87 (2)	2.28 (2)	2.7262 (19)	111.8 (15)
N1—H1*B*⋯O4^iv^	0.88 (2)	2.15 (2)	2.917 (2)	145.5 (16)

Symmetry codes: (i) 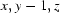; (ii) 
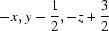
; (iii) 
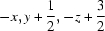
; (iv) 
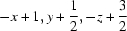
.

**Fig. 1.**
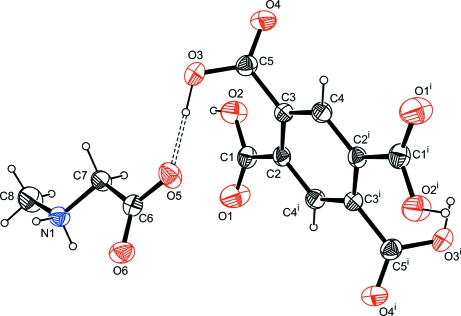
View of the title compound (I). Displacement ellipsoids are drawn at the 50% level. H atoms are represented as small spheres of arbitrary radii. H bond is represented as dashed line. [Symmetry code: (i) 1-x, 1-y, 2-z].

**Fig. 2.**
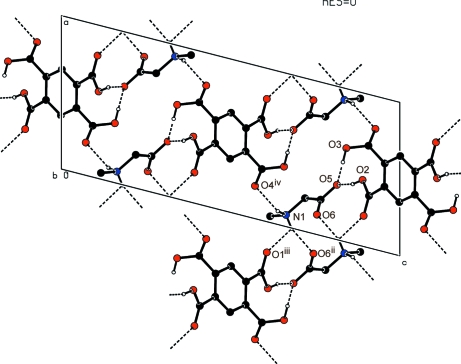
Packing view of the title compound down the b axis. Hydrogen bonds are depicted as dashed lines. H atoms not involved in hydrogen bondings have been omitted for clarity. [Symmetry codes: (ii) -x, y-1/2, -z+3/2; (iii) -x, y+1/2, -z+3/2; (iv) -x+1, y+1/2, -z+3/2]

## supplementary crystallographic information

### Comment

1,2,4,5-benzenetetracarboxylic acid (pyromellitic) is frequently chosen as a building block for crystal engineering due to its predictable properties and interesting supramolecular properties: It has provided three-dimensional porous networks (Yaghi *et al.*, 1997), host–guest systems (Arora & Pedireddi, 2003), mixed metallic systems (Rochon & Massarweh, 2001) and complex magnetic behaviours (Kumagai *et al.*, 2003). In an attempt to synthesize a low dimensional compound with copper, 1,2,4,5-benzenetetracarboxylic acid and sarcosine (as an auxiliary ligand), we have obtained the title compound, (I).

The midpoint of the acidic molecule lies on an inversion centre thus these molecules exhibit a C_i_ symmetry (Fig. 1). All four carboxylic groups retain the hydrogen atom and rotate around the C—C bond. Torsion angles O1—C1—C2—C4 24.1 (3)° and C4—C3—C5—O4 61.6 (2)° show different degrees of rotation. Sarcosine (*N*-methyl-glycine) crystallizes in the zwitterionic form with the amine group protonated and the carboxylic group deprotonated. The molecule when viewed along the C6—C7 bond shows the oxygen atoms anti to each other and the nitrogen atom synperiplanar to O6 [O6—C6—C7—N1 4.9 (2)°]. There is an extensive three-dimensional newtork of hydrogen bonds linking the molecules. Sarcosine molecules are assembled in chains *via* the N1—H1A···O6 bond (Table 1), running along the *b* axis. The chains are all interconnected through the remaining H bonds, since each sarcosine molecule is H-bonded to four benzenetetracarboxylic neighbours, (Fig. 2).

### Experimental

0. 5 mmol of copper hydroxyfluoride were added to a 20 ml warmed ethanolic solution containing 1.5 mmol of 1,2,4,5-benzenetetracarboxylic acid and 1.5 mmol of sarcosine. After a few weeks, transparent, colourless crystals could be isolated from the solution.

### Refinement

H atoms coordinates were located from a difference Fourier map and refined freely. The *U*_iso_(H) were restrained to be 1.2*U*_eq_(parent atom).

### Crystal data

**Table:**

C_10_H_6_O_8_·2C_3_H_7_NO_2_	*F*_000_ = 452
*M**_r_* = 432.34	*D*_x_ = 1.524 Mg m^−^^3^
Monoclinic, *P*2_1_/*c*	Mo *K*α radiation λ = 0.71073 Å
Hall symbol: -P 2ybc	Cell parameters from 2870 reflections
*a* = 8.8894 (3) Å	θ = 2.4–23.7º
*b* = 5.4118 (2) Å	µ = 0.13 mm^−^^1^
*c* = 20.2205 (7) Å	*T* = 293 (2) K
β = 104.388 (2)º	Prism, colourless
*V* = 942.25 (6) Å^3^	0.47 × 0.10 × 0.07 mm
*Z* = 2	

### Data collection

**Table:**

Bruker APEX CCD area-detector diffractometer	2351 independent reflections
Radiation source: fine-focus sealed tube	1643 reflections with *I* > 2σ(*I*)
Monochromator: graphite	*R*_int_ = 0.047
*T* = 293(2) K	θ_max_ = 28.4º
φ and ω scans	θ_min_ = 2.1º
Absorption correction: multi-scan(SADABS; Sheldrick, 2000)	*h* = −11→11
*T*_min_ = 0.915, *T*_max_ = 0.998	*k* = −7→7
16656 measured reflections	*l* = −27→27

### Refinement

**Table:**

Refinement on *F*^2^	Secondary atom site location: difference Fourier map
Least-squares matrix: full	Hydrogen site location: inferred from neighbouring sites
*R*[*F*^2^ > 2σ(*F*^2^)] = 0.041	Only H-atom coordinates refined
*wR*(*F*^2^) = 0.115	*w* = 1/[σ^2^(*F*_o_^2^) + (0.056*P*)^2^ + 0.2509*P*] where *P* = (*F*_o_^2^ + 2*F*_c_^2^)/3
*S* = 1.01	(Δ/σ)_max_ < 0.001
2351 reflections	Δρ_max_ = 0.25 e Å^−^^3^
166 parameters	Δρ_min_ = −0.21 e Å^−^^3^
Primary atom site location: structure-invariant direct methods	Extinction correction: none

### Special details

**Table:**

Geometry. All e.s.d.'s (except the e.s.d. in the dihedral angle between two l.s. planes) are estimated using the full covariance matrix. The cell e.s.d.'s are taken into account individually in the estimation of e.s.d.'s in distances, angles and torsion angles; correlations between e.s.d.'s in cell parameters are only used when they are defined by crystal symmetry. An approximate (isotropic) treatment of cell e.s.d.'s is used for estimating e.s.d.'s involving l.s. planes.
Refinement. Refinement of *F*^2^ against ALL reflections. The weighted *R*-factor *wR* and goodness of fit *S* are based on *F*^2^, conventional *R*-factors *R* are based on *F*, with *F* set to zero for negative *F*^2^. The threshold expression of *F*^2^ > σ(*F*^2^) is used only for calculating *R*-factors(gt) *etc*. and is not relevant to the choice of reflections for refinement. *R*-factors based on *F*^2^ are statistically about twice as large as those based on *F*, and *R*- factors based on ALL data will be even larger.

### Fractional atomic coordinates and isotropic or equivalent isotropic displacement parameters (Å^2^)

**Table:**

	*x*	*y*	*z*	*U*_iso_*/*U*_eq_	
O1	0.19992 (15)	0.1100 (3)	0.89313 (8)	0.0531 (4)	
O2	0.43230 (15)	−0.0346 (2)	0.88997 (7)	0.0415 (4)	
H2	0.389 (3)	−0.163 (4)	0.8612 (11)	0.050*	
C1	0.33933 (19)	0.1205 (3)	0.90904 (8)	0.0291 (4)	
C2	0.42466 (17)	0.3192 (3)	0.95451 (8)	0.0236 (3)	
C3	0.57683 (17)	0.3865 (3)	0.95565 (8)	0.0239 (3)	
C4	0.65079 (18)	0.5655 (3)	1.00139 (8)	0.0256 (3)	
H1	0.758 (2)	0.621 (3)	1.0018 (9)	0.031*	
C5	0.66506 (19)	0.2833 (3)	0.90749 (8)	0.0284 (4)	
O4	0.78636 (14)	0.1742 (3)	0.92796 (7)	0.0418 (4)	
O3	0.61224 (16)	0.3342 (3)	0.84210 (6)	0.0382 (3)	
H3	0.515 (2)	0.430 (4)	0.8318 (11)	0.046*	
O5	0.35723 (14)	0.5924 (2)	0.81422 (6)	0.0377 (3)	
O6	0.12981 (14)	0.7404 (2)	0.75521 (6)	0.0365 (3)	
C6	0.23381 (18)	0.5845 (3)	0.76612 (8)	0.0261 (4)	
C7	0.21727 (19)	0.3602 (3)	0.71983 (9)	0.0284 (4)	
H7A	0.205 (2)	0.202 (4)	0.7448 (9)	0.034*	
H7B	0.310 (2)	0.343 (3)	0.7009 (9)	0.034*	
N1	0.07624 (17)	0.3848 (3)	0.66375 (8)	0.0283 (3)	
H1A	−0.004 (2)	0.404 (4)	0.6805 (10)	0.034*	
H1B	0.087 (2)	0.516 (4)	0.6393 (10)	0.034*	
C8	0.0406 (3)	0.1733 (4)	0.61676 (12)	0.0437 (5)	
H8A	−0.047 (3)	0.212 (4)	0.5816 (12)	0.052*	
H8B	0.123 (3)	0.159 (4)	0.5931 (11)	0.052*	
H8C	0.033 (3)	0.037 (5)	0.6395 (12)	0.052*	

### Atomic displacement parameters (Å^2^)

**Table:**

	*U*^11^	*U*^22^	*U*^33^	*U*^12^	*U*^13^	*U*^23^
O1	0.0296 (7)	0.0604 (10)	0.0672 (10)	−0.0132 (6)	0.0080 (6)	−0.0350 (8)
O2	0.0364 (7)	0.0329 (7)	0.0517 (8)	−0.0029 (6)	0.0042 (6)	−0.0215 (6)
C1	0.0313 (9)	0.0282 (9)	0.0271 (8)	−0.0057 (7)	0.0061 (7)	−0.0040 (7)
C2	0.0250 (8)	0.0231 (8)	0.0213 (8)	−0.0009 (6)	0.0030 (6)	−0.0020 (6)
C3	0.0240 (7)	0.0247 (8)	0.0227 (8)	0.0016 (6)	0.0054 (6)	−0.0013 (6)
C4	0.0227 (7)	0.0282 (9)	0.0255 (8)	−0.0026 (6)	0.0052 (6)	−0.0021 (7)
C5	0.0275 (8)	0.0283 (9)	0.0298 (9)	−0.0015 (7)	0.0079 (7)	−0.0059 (7)
O4	0.0333 (7)	0.0487 (8)	0.0437 (8)	0.0126 (6)	0.0105 (6)	−0.0056 (6)
O3	0.0410 (7)	0.0487 (8)	0.0271 (7)	0.0070 (6)	0.0128 (5)	−0.0032 (6)
O5	0.0416 (7)	0.0326 (7)	0.0321 (7)	0.0032 (6)	−0.0034 (5)	−0.0080 (5)
O6	0.0374 (7)	0.0330 (7)	0.0393 (7)	0.0073 (6)	0.0098 (6)	−0.0068 (6)
C6	0.0295 (8)	0.0253 (8)	0.0242 (8)	−0.0017 (7)	0.0079 (6)	−0.0009 (7)
C7	0.0292 (9)	0.0258 (9)	0.0274 (9)	0.0031 (7)	0.0019 (7)	−0.0028 (7)
N1	0.0255 (7)	0.0285 (8)	0.0292 (8)	0.0006 (6)	0.0036 (6)	−0.0038 (6)
C8	0.0486 (12)	0.0370 (11)	0.0390 (11)	0.0027 (10)	−0.0015 (10)	−0.0142 (9)

### Geometric parameters (Å, °)

**Table:**

O1—C1	1.202 (2)	O5—C6	1.273 (2)
O2—C1	1.302 (2)	O6—C6	1.230 (2)
O2—H2	0.93 (2)	C6—C7	1.518 (2)
C1—C2	1.493 (2)	C7—N1	1.473 (2)
C2—C4^i^	1.390 (2)	C7—H7A	1.02 (2)
C2—C3	1.396 (2)	C7—H7B	0.995 (19)
C3—C4	1.387 (2)	N1—C8	1.471 (2)
C3—C5	1.502 (2)	N1—H1A	0.87 (2)
C4—C2^i^	1.390 (2)	N1—H1B	0.88 (2)
C4—H1	0.997 (18)	C8—H8A	0.94 (2)
C5—O4	1.209 (2)	C8—H8B	0.97 (2)
C5—O3	1.318 (2)	C8—H8C	0.88 (2)
O3—H3	0.98 (2)		
			
C1—O2—H2	118.4 (14)	O5—C6—C7	115.53 (14)
O1—C1—O2	125.37 (16)	N1—C7—C6	109.64 (13)
O1—C1—C2	122.06 (16)	N1—C7—H7A	106.5 (11)
O2—C1—C2	112.57 (14)	C6—C7—H7A	112.0 (10)
C4^i^—C2—C3	119.49 (14)	N1—C7—H7B	109.9 (11)
C4^i^—C2—C1	117.78 (14)	C6—C7—H7B	110.5 (11)
C3—C2—C1	122.70 (14)	H7A—C7—H7B	108.2 (15)
C4—C3—C2	119.36 (14)	C8—N1—C7	115.63 (15)
C4—C3—C5	117.05 (14)	C8—N1—H1A	106.3 (13)
C2—C3—C5	123.53 (14)	C7—N1—H1A	109.5 (13)
C3—C4—C2^i^	121.14 (14)	C8—N1—H1B	107.4 (13)
C3—C4—H1	120.5 (10)	C7—N1—H1B	108.1 (13)
C2^i^—C4—H1	118.3 (11)	H1A—N1—H1B	109.8 (18)
O4—C5—O3	120.72 (15)	N1—C8—H8A	108.6 (14)
O4—C5—C3	121.66 (15)	N1—C8—H8B	108.0 (14)
O3—C5—C3	117.36 (14)	H8A—C8—H8B	103.5 (18)
C5—O3—H3	113.4 (12)	N1—C8—H8C	110.2 (16)
O6—C6—O5	125.48 (16)	H8A—C8—H8C	115 (2)
O6—C6—C7	118.98 (15)	H8B—C8—H8C	111 (2)
			
O1—C1—C2—C4^i^	24.1 (3)	C5—C3—C4—C2^i^	176.64 (15)
O2—C1—C2—C4^i^	−155.56 (15)	C4—C3—C5—O4	61.6 (2)
O1—C1—C2—C3	−157.90 (18)	C2—C3—C5—O4	−121.1 (2)
O2—C1—C2—C3	22.4 (2)	C4—C3—C5—O3	−112.73 (18)
C4^i^—C2—C3—C4	0.7 (3)	C2—C3—C5—O3	64.5 (2)
C1—C2—C3—C4	−177.23 (15)	O6—C6—C7—N1	4.9 (2)
C4^i^—C2—C3—C5	−176.47 (15)	O5—C6—C7—N1	−175.62 (15)
C1—C2—C3—C5	5.5 (3)	C6—C7—N1—C8	−176.75 (17)
C2—C3—C4—C2^i^	−0.8 (3)		

Symmetry codes: (i) −*x*+1, −*y*+1, −*z*+2.

### Hydrogen-bond geometry (Å, °)

**Table:**

*D*—H···*A*	*D*—H	H···*A*	*D*···*A*	*D*—H···*A*
O2—H2···O5^ii^	0.93 (2)	1.61 (2)	2.5216 (18)	166 (2)
O3—H3···O5	0.98 (2)	1.62 (2)	2.6026 (18)	179 (2)
N1—H1A···O6^iii^	0.87 (2)	2.11 (2)	2.854 (2)	143.4 (17)
N1—H1A···O1^iv^	0.87 (2)	2.28 (2)	2.7262 (19)	111.8 (15)
N1—H1B···O4^v^	0.88 (2)	2.15 (2)	2.917 (2)	145.5 (16)

Symmetry codes: (ii) *x*, *y*−1, *z*; (iii) −*x*, *y*−1/2, −*z*+3/2; (iv) −*x*, *y*+1/2, −*z*+3/2; (v) −*x*+1, *y*+1/2, −*z*+3/2.
